# On Cutaneous Diseases

**Published:** 1801-10

**Authors:** 


### *On CUTANEOUS DISEASES.*

GREAT improvements in the practice of Medicine and Surgery generally proceed from the concurrent labours of many; there are, however, inftances where individuals, favourites of Apollo, who have devoted long and continued attention to the inveftigation of particular fubjects, have merited the whole honour of their improvements. Of the dead, the examples are numerous, from Hippocrates to Hunter; of the living, our Journal already contains two memorable inftances, Dr. JENNER'S introduction of the Vaccine Inoculation, and Dr. JACKSON'S* inveftigation of the nature and caufes of Idiopathic Fever.—We now proceed to a third, where the labours and difficulties have been greater than in the preceding, and from which the Profeffion will not derive lefs advantage. The public expectation has rarely been raifed fo high, or fo completely gratified, as far as the Work has proceeded, as by the DESCRIPTION AND TREATMENT OF CUTANEOUS DISEASES, by ROBERT WILLAN, M. D. F. A. S. 4to. about 110 pages each Order, illuftrated with coloured plates. London, Johnfon.

The term "Cutaneous Difeafes" is familiar enough to every practitioner, and fome of them occur fo frequently that moft people can diftinguifh them with fufficient eafe and certainty; but we believe there are very few, even of Profeffors, who have any fyftematic knowledge of the whole fubject; and we know that what was to be found in books, before the prefent publication, was too confufed and imperfect to repay the time of reading them. A wildernefs fo completely uncultivated did Dr. W. find difeafes of the fkin, that he was obliged to adopt new principles of claffification, and almoft to create a new language, before he could commence his labours. We do not mean that he *rejected* old and appropriate names when they could be found, but that he was obliged to infert many new ones; and to give, what they never had before, a precife meaning to the old ones. It is obvious, therefore, that previous to our commencing any intelligible account of this elaborate work, we muft give an explanation of the fenfe in which Dr. Willan ufes the terms employed in his definitions and defcriptions of thefe complaints. It may be proper, however, to premife, that the Author propofes to diftinguifh all thefe affections of the fkin, whether attended with conftitutional derangement or not, by obvious external marks, which are reprefented by coloured plates.

---

* Vol. i. p. 225.

### DEFINITIONS.

1. Scurf (furfura); ſmall exfoliations of the cuticle, which take place after ſome eruptions on the ſkin; a new cuticle being formed underneath during the exfoliation.

2. Scale (Squama); a Lamina of morbid cuticle, hard, thickened, whitiſh, and opake. Scales have at firſt the figure and extent of the cuticular lozenges; but afterwards often increaſe into irregular layers, denominated crusts. Both ſcales and cruſts repeatedly fall off, and are reproduced in a ſhort time.

3. Scab; a hard ſubſtance covering ſuperficial ulcerations, and formed by a concretion of the fluid diſcharged from them.

4. Stigma; a ſmall red ſpeck in the ſkin, occaſioning no elevation of the cuticle. Stigmata are generally diſtinct or apart from each other. They ſometimes aſſume a livid colour; and are then termed petechiæ.

5. Papula; a very ſmall and acuminated elevation of the cuticle, with an inflamed baſe, not containing a fluid, not tending to ſuppuration. The duration of Papulæ is uncertain; but they terminate, for the moſt part, in ſcurf.

6. Rash (Exanthema); conſiſts of red patches on the ſkin, variouſly figured, in general confluent, and diffuſed irregularly over the body, leaving interſtices of a natural colour. Portions of the cuticle are often elevated in a raſh, but the elevations are not acuminated. The eruption is uſually accompanied with a general diſorder of the conſtitution, and terminates in a few days by cuticular exfoliations.

7. Macula; a permanent diſcolouration of ſome portion of the ſkin, often with a change of its texture, but not connected with any diſorder of the conſtitution.

8. Tubercle; a hard, ſuperficial tumour, circumſcribed, and permanent; or proceeding very ſlowly to ſuppuration.

9. Vesicle (Bulla); an elevation of the cuticle, of a large ſize, irregularly circumſcribed, and containing a tranſparent watery fluid. Veſicles with a dark red or livid-coloured baſe are uſually denominated phlyctænæ.

10. Pustule; an elevation of the cuticle, ſometimes globate, ſometimes conoidal in its form, and containing pus, or a lymph which is in general diſcoloured. Puſtules are various in their ſize, but the diameter of the largeſt ſeldom exceeds two lines.

There are many different kinds of puſtules properly diſtinguiſhed in medical authors by ſpecific appellations; as,

1. Phlyzacium; a puſtule containing pus, and raiſed on a hard a hard, circular, inflamed bafe, of a vivid red colour. It is fucceeded by a thick, hard, dark-coloured fcab.

2. PSYDRACIUM; a minute puftule, irregularly circumfcribed, producing but a flight elevation of the cuticle, and terminating in a laminated fcab. Many of thefe puftules ufually appear together, and become confluent. When mature, they contain pus; and after breaking, difcharge a thin watery humour.

3. ACHOR; a puftule of intermediate fize between the two foregoing, which contains a ftraw-coloured fluid, having the appearance, and nearly the confiftence, of ftrained honey. It appears moft frequently about the head, and is fucceeded by a dull white, or yellowifh fcab.

Puftules of this kind, when fo large as nearly to equal the fize of Phlyzacia, are termed CERIA or FAVI; being fucceeded by a yellow, femi-tranfparent, and fometimes cellular fcab, like a honey-comb.

4. PHLYCTIS; a fmall puftule with a circular bafe flightly inflamed, containing a lymph which is fometimes clear and pellucid, but more frequently whitifh like whey, or pearl-coloured. The puftule terminates in a laminated fcab.

Under this head may be ranked the puftules denominated by authors Hydroa or Hidroa, Boa, Sudamina, and miliary puftules.

"I propofe," fays Dr. W. "to arrange Cutaneous Difeafes in feven orders, to be characterized by the different appearances of Papulæ, Scales, Rafhes, Veficles, Puftules, Tubercles, and Maculæ. By comparing together the 2d, 5th, 6th, 7th, 8th, 9th, and 10th definitions, the diftinguifhing characters of each order may be readily underftood, and will be farther illuftrated in treating of the orders refpectively.

Thefe Orders, with the Generic Diftinctions comprifed under each, are as follow:

ORD. I. Strophulus (Red Gum, Tooth Eruption, &c.)
Lichen (Spring Eruption, Scorbutic Pimples, &c.)
Prurigo (Gratelle, or Univerfal Itching of the Skin

ORD. II. Lepra (Leprofy of the Greeks)

Pforiafis (Dry or Scaly Tetter)

Pityriafis (Dandriff)

Icthyofis (Fifh Skin)

ORD. III. Rubeola (Meafles)

Scarlatina (Scarlet-Fever)

Urticaria (Nettle-Rafh)

Rofeola (Summer-Rafh, or Rofe Rafh)

Purpura (Purple or Scorbutic Rash)
Erythema Red Rash)
ORD. IV. Erysipelas (St. Anthony's Fire)
Pemphigus (Vesicular Fever)
Pompholyx (Water Blebs)
Herpes (Ringworm, Shingles, Wildfire, &c.)
Vacciola (Cow Pox)
Varicella (Chicken Pox)
Miliaria (Miliary Eruptions)
Eczema (Heat Eruption)
Aphthæ (Thrush)
ORD. V. Impetigo (Running Scab)
Ecthyma (Ulcerated Tetter)
Variola (Small Pox
Scabies (Itch)
Porrigo (Scald Head, &c.)
ORD. VI. Phyma (Boils, Carbuncles, &c.)
Verruca (Warts)
Acne (Stone Pock; red, tuberculated Face, &c.)
Lupus, or Noli me Tangere
Elephantiasis (Arabian Leprosy)
Frambæsia (Yaws)
ORD. VII. Ephelis (Sun Spots)
Nævus
Spilus, Moles, and other original Marks.

The first Order is thus characterised:

ORDER I.—PAPULÆ.

"Papulæ may be considered as enlargements of the Papillæ of the skin, occasioned by a strong determination of blood to them, sometimes attended with a degree of inflammation. The small Papillæ thus enlarged elevate the cuticle immediately above them, and appear red. A slight effusion of Lymph often takes place in these circumstances, and gives a pustular form to several of the Papulæ: but the fluid is re-absorbed without breaking the cuticle. The term Papula is employed by medical authors in various significations. Marcellus and Scribonius Largus extend it to express herpetic pustules, achores, and vari. Pliny includes under this title, wheals, miliary pustules, and petechiæ. Prof. Lorry and others also apply it to wheals and superficial tubercles, or pustules of various kinds. Plenck, in his Doctrina de Morbis Cutaneis, Class 5, has defined Papulæ as follows; Sunt tumores exigui sed duri qui vel resolvuntur, vel ex Apice quid humidi ejiciunt et dein desquammantur. Yet he comprehends in the class, several affections, not properly answering his specification of it, nor at all related to each other, as Vari, Grutum seu Milium, Herpes, Cutis Anserina, Tuberculum,

Tuberculum, Phygethlon, Lepra, Elephantiafis. It appears therefore that fome limitation of the fenfe of this term is neceffary. In defining it (Def. 5.) I have followed the authority of Sauvage and Linnæus. If that definition be ftrictly adhered to, few genera will be found belonging to this order. I have only obferved three, which merit particular notice, Strophulus, Lichen, and Prurigo.

"I. The STROPHULUS is a papulous eruption, peculiar to infants, and exhibiting a variety of forms, which may be defcribed under the titles of Strophulus Intertinctus, Strophulus Albidus, Strophulus Confertus, Strophulus Volaticus, Strophulus Candidus.

"1. Strophulus Intertinctus, ufually called, the Red Gum, and by the French, Efflorescence Benigne. The Papulæ charácterifing this affection, rife fenfibly above the level of the cuticle, are of a vivid red colour, and commonly diftinct from each other. Their number and extent varies much in different cafes. They appear, moft conftantly, on the cheeks, fore-arm, and back of the hand; but are fometimes diffufed over the whole body. The papulæ are, in many places, intermixed with ftigmata (Def. 5.), and often with red patches of a larger fize, which do not however occafion any elevation of the cuticle. A child's fkin thus variegated, fomewhat refembles a piece of red printed linen: and hence this eruption was formerly denominated the Red-Gown, a term which is ftill retained in feveral counties of England, and may be found in old dictionaries. Medical writers have changed the original word for one of a fimilar found, but not more fignificant."

[ To be continued. ]

